# Exchanging Catheters Over a Single Transseptal Sheath During Left Atrial Ablation is Associated with a Higher Risk for Silent Cerebral Events

**DOI:** 10.1016/s0972-6292(16)30795-1

**Published:** 2014-10-06

**Authors:** Thomas Deneke, Karin Nentwich, Rainer Schmitt, Georgios Christhopoulos, Joachim Krug, Luigi Di Biase, Andrea Natale, Atilla Szollosi, Andreas Mugge, Patrick Muller, Johannes W Dietrich, Dong-In Shin, Sebastian Kerber, Anja Schade

**Affiliations:** 1 Heart Center Bad Neustadt, Clinic for invasive Electrophysiology, Bad Neustadt, GER; 2 Ruhr-University Bochum, Bochum, GER; 3 Department of Radiology, Bad Neustadt, GER; 4 Texas Cardiac Arrhythmia Institute at St. David`s Medical Center, Austin, USA; 5 Department of Biomedical Engineering, University of Texas, Austin, USA; 6 Department of Cardiology, University of Foggia, Foggia, Italy

**Keywords:** Silent cerebral lesions, atrial fibrillation ablation, magnetic resonance imaging

## Abstract

**Background:**

Silent cerebral events (SCE) have been identified on magnetic resonance imaging (MRI) in asymptomatic patients after atrial fibrillation (AF) ablation. Procedural determinants influencing the risk for SCE still remain unclear.

**Objective:**

Comparing the risk for SCE depending on exchanges of catheters (ExCath) over a single transseptal sheath.

**Methods:**

88 Patients undergoing pulmonary vein isolation (PVI) only ablation using either single-tip or balloon-based technique underwent pre- and post-ablation cerebral MRI. Ablations were either performed with double transseptal access and without exchanging catheters over the transseptal sheaths (group 1: no ExCath) or after a single transseptal access and exchanges of therapeutic and diagnostic catheters (group 2: ExCath). Differences in regard to SCE rates were analyzed. Multivariate analysis was performed to identify factors related to the risk for SCE.

**Results:**

Included patients underwent PVI using single tip irrigated radiofrequency in 41, endoscopic laser balloon in 27 and cryoballoon in 20 cases. Overall SCE were identified in 23 (26%) patients. In group 1 (no ExCath; N=46) 6 patients (13%) and in group 2 (N=42) 17 patients (40%) had documented SCE (p=0.007). The applied ablation technology did not affect SCE rate. In multivariate analysis age (OR 1.1, p=0.03) and catheter exchanges over a single transseptal sheath (OR 12.1, p=0.007) were the only independent predictors of a higher risk for SCE.

**Conclusions:**

Exchanging catheters over a single transseptal access to perform left atrial ablation is associated with a significantly higher incidence of SCE compared to an ablation technique using different transseptal accesses for therapeutic and diagnostic catheters.

## Introduction

Cerebral infarction is a rare but devastating complication of ablation procedures to treat atrial fibrillation (AF). But symptomatic cerebral ischemia may only be the tip of the iceberg as recent studies have identified cerebral lesions in asymptomatic patients after pulmonary vein isolation (PVI) undergoing post-ablation brain magnetic resonance imaging (MRI) [1-4]. Incidences of silent cerebral events (SCE) appear to depend on ablation technology [2-6] and may be as high as 41% [4]. The clinical relevance of these post-ablation SCE still remains unclear. Different predictors of SCE have been identified but so far different procedural steps of the ablation procedure have not been evaluated in detail in regard to their potential risk [3-8]. Exchanges of catheters over a single transseptal sheath may involve a higher risk for embolic events due to manipulation of the sheath introducing air or thrombi. The present study evaluates the risk for SCE comparing left atrial ablation techniques using a single transseptal access and exchanging diagnostic and therapeutic catheters to a technique using two transseptal accesses, one for each catheter.

## Methods

Patients were eligible if undergoing pulmonary vein isolation (PVI) alone for paroxysmal or short-lasting persistent AF (< 3 months sustained AF) using either single-tip radiofrequency ablation (RF) or balloon-based technologies (cryo-balloon or endoscopic assisted laser-balloon ablation) and if willing and able to undergo cerebral MRI. Patients were categorized as either having paroxysmal or persistent AF based on current guidelines. All patients underwent MRI scanning one day before and 1 to 4 days after PVI. Baseline and procedural patient data was collected including procedure duration (from puncture to sheaths out), total ablation duration, minimal and mean procedural activated clotting time (ACT), periprocedural anticoagulation management (continued oral anticoagulation, bridging using low-molecular weight heparin), echocardiographic evaluation (left atrial enlargement was defined as left atrial dimensions being out of normal values, left ventricular ejection fraction), intraprocedural cardioversion or occurrence or persistence of AF.

All patients were off anticoagulation at the day of the procedure and were bridged using low-molecular weight heparin (half body-weight adjusted dose at day of the procedure and full body-weight adjusted dose afterwards until adequate oral anticoagulation was achieved). Warfarin medication or novel oral anticoagulants were administered on day 1 after the ablation procedure if access site was clinically blunt.

All patients gave informed consent and procedures were performed after exclusion of intracardiac thrombus using transesophageal imaging. Procedures were performed under conscious sedation using propofol and via transseptal access to the left atrium in standard technique.

In both groups ablations were started only after the ACT reached ≥ 300sec. Intraprocedurally, ACTs were controlled every 20 minutes for the course of the procedure. Minimum ACT was documented for each procedure. All transseptal sheaths were flushed continuously with a rate of 150ml/hr. In cases with exchanges of catheters over a single transseptal sheath blood was aspired after taking catheters out and high-volume flushed (999ml/hr) until the next catheter was introduced. Care was taken to avoid any introduction of air into the transseptal apparatus. Ablation procedures were performed using 3 different technologies as previously described [7,9,11]. Patient selection for each ablation technique was based on operators' preference.

A neurological evaluation by board-certified neurologists was performed the day of post-ablation MRI. In all cases only PVI was performed and no additional left atrial ablations were performed.

1. Irrigated tip radiofrequency (irrRF) ablation was performed using CARTO3 (Biosense Webster, Diamond Bar, USA) or NavX (St. Jude Medical, St. Paul, USA) electroanatomic mapping system. Either single transseptal access (with one transseptal sheath and exchange of diagnostic and ablation catheters over the sheath) or double transseptal access (two different transseptal sheaths for the diagnostic and the ablation catheter) was performed. The circular diagnostic PV mapping catheter was used to create a 3-dimensional shell of the left atrium and prior computed tomography images were integrated by merging. In all cases a steerable transseptal sheath (AGILIS™, St. Jude Medical, St. Paul, USA) was used for the ablation catheter and isolation of the ipsilateral PVs was performed en-block in a point-by-point fashion. Ablation was performed using a maximum of 35Watts with a target temperature of 43ºC and a maximum irrigation rate of 30ml/min.

2. Ablation using the HeartLight™ Endoscopic Ablation system (EAS) (CardioFocus, Marlborough, USA) was performed after either a single or a double transseptal access. A CMC was used to confirm bidirectional PV block (either as an exchange with the laser balloon system in patients with single transseptal approach or via the second transseptal sheath). Endoscopically assisted laser-balloon ablations for PVI were performed as previously described [11-13]. Ablations were performed with the 30º-angle Argon laser to produce overlapping lesions encircling the PV. Each ablation was performed for 20 to 30 seconds using 5.5 to 12Watt energy as estimated by the operator. After completion of encircling (visually guided) the 10-pole circular mapping catheter was used to identify residual PV-conduction or block. If residual conduction was seen, further ablations were performed in that segment of the PV. In case of a single transseptal approach PV-conduction was tested after singular encircling of each PV exchanging catheters. If residual conduction was found, the catheters had to be exchanged at least twice to perform further ablations and check afterwards.

3. The ArcticFront™ (Medtronic, Minneapolis, USA) cryo-balloon (CB) was used for PVI. A single or double transseptal access (12 F FlexCath™, Medtronic, Minneapolis, USA) was performed and either a CMC was used for pre- and post-ablation evaluation (as an exchange of catheters or over two distinct accesses) or the Achieve™ (Medtronic, Minneapolis, USA) catheter was used as a guide-wire for the CB catheter and for diagnosis of PV-block (no exchange of catheters). At each PV at least two freezes lasting 300 seconds were carried out. In case of usage of the Achieve™ online monitoring of PV conduction was possible and further CB applications were performed directly if persistent PV conduction was found. If a single transseptal approach using a conventional circular mapping catheter was carried out, PV conduction was tested after performing 2 CB applications in each PV and the catheters had been exchanged. Further CB applications were performed if persistent PV conduction was found after exchanging the catheters again. No additional focal touch up ablations were performed.

### Exchange of Catheters over a single transseptal sheath

*Group 1:* No exchange of catheters (no ExCath) was defined as two transseptal accesses using two distinct transseptal sheaths for the CMC and the ablation device (two separate transseptal sheaths) or if the Achieve™ catheter was used through the inner lumen of the CB.

*Group 2:* Exchange of catheters over a single transseptal sheath (ExCath) was defined as exchanging CMC and ablation device at least twice (CMC before and after ablation).

In all patients the endpoint of PVI was confirmed as bidirectional PV block including entrance block (loss of PV potentials) and exit block (when pacing the PV from distal the isolation site).

### Cerebral Magnetic Resonance Imaging (MRI)

MRI scans were independently evaluated by two board-certified radiologists experienced in the diagnosis of brain MRI scans blinded for the ablation technology used.

Brain MRI was 1 to 3 days after ablation and during follow-up using a 1.5-T scanner (Magnetom Avanto, Siemens, Erlangen, Germany). The imaging protocol for all images consisted of a transaxial T2-weighted, fluid-attenuated inversion recovery (FLAIR) sequence (TI 2500ms, TR 8500ms, TE 112ms, slice thickness 5.0mm, in-plane resolution 1.6x0.9x5.0mm^3^, flip angle 150º) and a transaxial diffusion-weighted echo-planar imaging (DWI) sequence (TR 3300ms, TE 99ms, in-plane resolution 1.9x1.3x5.0mm^3^). For each DWI sequence, the apparent diffusion coefficient (ADC) map was obtained. Additionally, a sagital T1-weighted spin-echo sequence (TR 400ms, TE 13ms, in plane resolution 1.2x0.9x5.0mm^3^, flip angle 70º) was acquired for assessing brain anatomy and for ruling out haemorrhaging conversion of the SCEs.

**Figure 1:**
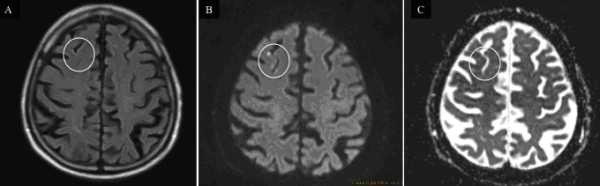
Post-ablation MRI with three distinct SCEs (white circle) not identified on FLAIR (A) but documented as hyperintense lesions in DWI (B) and corresponding hypointense lesion in ADC-map (C) defining SCE.

SCE was defined as a hyperintense lesion in DWI scans with a corresponding hypo-attenuated area in the ADC map. The FLAIR sequence was not used to define SCE (Figure 1). Occurrence, number and size of SCEs were documented for each individual patient. Proton density (PD)-scans were performed additionally in post-ablation follow up MRI to increase sensitivity concerning SCE.

### Statistics

Statistical analyses were carried out with custom S-scripts for the statistical environment R 2.10.1 for Mac OS X. Where not otherwise specified, data is presented as mean±standard error of the mean for normally distributed continuous data or median (interquartile range) for non-normally distributed data or percentages. Depending from the class of analyzed data and possible direction of causality, distributions were compared by means of multivariate logistic regression with forward selection, two sample t-test, Wilcoxons rank sum test or Pearson's Chi-squared test with Yates' continuity correction. For multivariate logistic regression variables were selected that differed with p<0.1 in a-priori Chi-squared test or Wilcoxons test and that did not correlate to procedure, respectively. A two-tailed p value of <0.05 was considered statistically significant in all tests.

## Results

A total of 88 patients were included (41 patients treated with IrrRF, 27 with EAS and 20 with CB) during the study period. 46 patients were ablated without exchange of catheters over a single transseptal sheath (group 1, no ExCath) and 42 with (group 2, ExCath) (baseline patient characteristics see Table 1).

### Post-ablation cerebral MRI

Brain MRI was performed at a median of 1 day after the procedure (1 to 4 days). 2 (2%) patients had old lacunar infarction and 35 (40%) had unspecific white matter lesions and glial scarring identified as leucencephalopathy.

No patient had new-onset neurological symptoms indicative of cerebral ischemia after the procedure. 23 patients (26%) had documented SCE (8 (20%) patients treated with IrrRF, 10 (37%) with EAS and 5 (25%) with CB). Overall 51 SCEs were identified (2.2/patient; diameter in between 1 to 11mm), 41 (80%) were 3mm or less in diameter, 9 (18%) in between 4 to 10mm and 1 (2%) was larger than 10mm in diameter (see Figure 1).

### Effect of ExCath

Baseline and procedural characteristics in relation to ExCath are displayed in Table 2. In Group 1 patients (no ExCath) irrRF was more common compared to EAS and CB. Mean ACT tended to be higher in group 1 patients but without statistical significance (p=0.05).

Incidence of SCE was significantly higher in group 2 patients (40%, 17/42) compared to those group 1 (13%, 6/46) (p=0.004) (Table 2). Also, number per patient (2.2±0.63 versus 2.7±0.14, p=0.005) and mean diameter of SCE (4±2mm vs. 5±04mm, p=0.45) differed significantly (Table 2).

### Baseline and procedural characteristics comparing patients with and without SCE

Comparative analysis of baseline characteristics is shown in Table 1. No patient had detected noticeable abnormalities on neurological evaluation.

Patients with SCE compared to those without were significantly older (69±8 vs. 59±12, p=0.04) and significantly more often ExCath was performed (40% versus 74%, p=0.007).

No difference in any other tested variable was identified comparing patients with and without SCE (Table 2).

### Comparison in between ablation technologies

**Figure 2:**
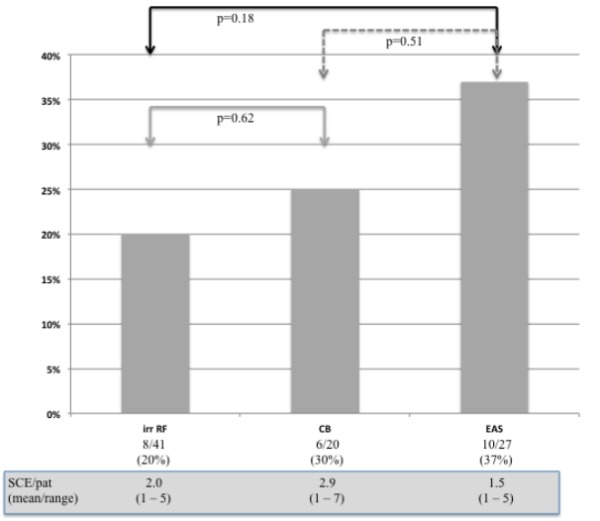
SCE rates and SCE/pat in the different ablation groups

Comparing incidence of SCE and number of SCE/pt in between different ablation technologies did not reveal significant differences: The incidence of SCE was 20% in IrrRF (8 out of 41), 30% in CB (6 out of 20) and 37% in EAS (10/27) patients ( Figure 2).

### Multivariate analysis of risk of SCE

In the multivariate analysis (Table 3) only age (p=0.03, OR 1.07) and ExCath (p=0.006, OR 12.93) were independent significant predictors of increased risk for post-ablation SCL (Table 3). ExCath was associated with a 3.1-times increased relative risk for SCL.

In multivariate analysis mode of ablation (irrRF, EAS, CB) did not affect SCE incidence.

## Discussion

The presented data indicates that exchanging catheters over a single transseptal sheath during left atrial ablations significantly influence the risk for acute SCE identified on post-ablation MRI. In addition, age was also associated (but not as strong) with an increased risk for SCE.

### Exchanges of catheters and risk for SCE

We identified a significantly higher incidence of SCE in patients with catheter exchanges over the transseptal sheath of 40% compared to 13% in the group of patients ablated without catheter exchanges. ExCath over a single transseptal sheath in our analysis was the strongest predictor for SCE associated with a 3.1-imes increased risk. Introducing and withdrawing a catheter over the transseptal sheath may lead to introduction of gas or thrombotic debris from within the transseptal sheath or the catheter. In addition, ExCath over a single transseptal sheath may influence the patency of the sheath's valve and may then lead to introduction of small air bubbles. In patients undergoing either single-tip irrRF ablation or balloon-based PVI the technique may include either having two transseptal sheaths (one for the ablation device and one for the circular mapping catheter) or only a single transseptal sheath with the need for exchanging diagnostic and therapeutic catheters. Further studies are needed to identify e.g. the number of exchanges that may be detrimental or may identify differences in between transsseptal sheath designs or size. In our study catheter exchanges were performed at least twice in group 2 patients. ExCath over a single transseptal sheath should be avoided in order to reduce the incidence of SCE.

Predictors, so far identified to impact SCE rates, are intraprocedural ACT [3,5,6], intraprocedural cardioversion [3,6], echocontrast on pre-ablation transesophageal echo [4,6] and ablation of sites with complex fractionated electrograms [6,14] as independent predictors of SCE. So far, data on all of these variables appears controversial [2-4,15] which may be related to the different MRI definitions (see below) or MRI technologies used. In our analysis using a high-sensitive MRI definition of SCE only older age and ExCath were independent predictors of SCE.

Recently a study on phased RF ablation using the PVAC catheters indicated that subtle changes of the procedural steps lead to a dramatic reduction in FLAIR-positive silent cerebral lesions [16]. Further studies need to identify steps of left atrial ablation procedures most likely to involve embolism and generation of SCE.

### SCE in post-ablation MRI

SCE detected on cerebral MRI have been identified as a potential embolic complication of left atrial ablation procedures. Incidence of detected SCE depends upon MRI technique and definition used. In our study we used a more sensitive ("new") definition of SCE characterized only by a hyperintensity on DWI-MRI and corresponding hypointense lesion on ADC-map. In contrast to other (earlier) studies we have not included the FLAIR sequence, which may misclassify some DWI hyperintense lesions as negative due to the timeline of FLAIR becoming positive (usually over the first 48 hours or longer). This may lead to undertection of (mostly smaller) silent cerebral events in up to 2/3 of lesions [17]. Schmidt et al. [8] using the same definition as in our study found comparable silent cerebral event rates (for IrrRF 24%, for Cryo ablation 18% and 24% for EAS ablation).

In summary, using a more sensitive MRI definition more patients with SCE are detected (higher incidences) and differences between technologies appear to be less pronounced.

So far, the exact mechanism of SCE generation remains unclear. Recent animal studies indicate that silent cerebral lesions usually dissolved on MRI within 4 days after occurrence although glial scar formation was detected on histopathology. Lesions detected on DWI and FLAIR in this study appear to present small asymptomatic strokes. The incidence of SCE may serve as a surrogate parameter for the embolic load of different left atrial ablation technologies and techniques. Whether different sizes of SCE represent different mechanisms (e.g. gaseous versus thrombotic embolism) and why and if lesions really remain asymptomatic using high precision neuropsychological testing needs to be further elucidated. So far no study has identified potential clinical sequel related to post-ablation SCE and their small number and size needs to be weighted against the high incidence and number of pre-existing white matter lesions related to the process of AF.

## Limitations

The present study evaluates SCE rates related to intraprocedural exchanges of left atrial catheters over a single transseptal sheath using a sensitive methodology. Although many baseline and procedural characteristics have been evaluated, other - so far unknown - factors may also play a role or may only become visible in larger patient population.

The number of exchanges of catheters over a single transseptal sheath and different sheath designs were not evaluated and further studies need to identify potential differences in regard to the sheath or technology used. It may be speculated that mismatches between catheter sizes and sheath diameter may also impact the risk for SCE. Furthermore, the effect of continued anticoagulation or novel oral anticoagulant during left atrial ablation was not studied in the present evaluation. Recent data indicates relevant differences in SCE incidences in between patients ablated on continued oral anticoagulation versus other protocols (Deneke et al., 2013, presented at the Annual Scientific Congress of the American Heart Association, Dallas). This was not tested in the present study using a prespecified periprocedural anticoagulation protocol.

## Conclusions

Risk for SCE appears to be multifactorial: As a new but modifiable determinant we identified exchanges of catheters over a single transseptal sheath as a strong factor involving a higher risk for SCE. When using two different catheters for PVI diagnostics and ablation different accesses (sheaths) should be used to reduce SCE rates.

SCE rates may serve as an indicator for the embolic risk of a left atrial ablation technology or procedure. Procedural and technological changes to minimize the incidence of SCE should be attempted.

## Figures and Tables

**Table 1:**
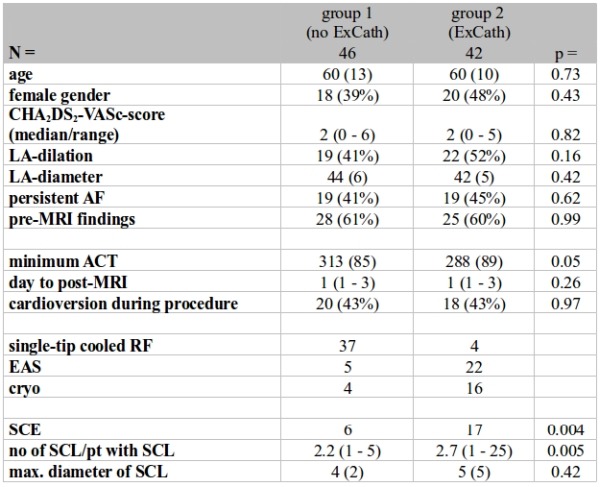
Baseline, procedural and outcome data comparing patients without exchanges of catheters of a single transseptal sheath (group 1) to those with exchangs of catheter (group 2)

**Table 2:**
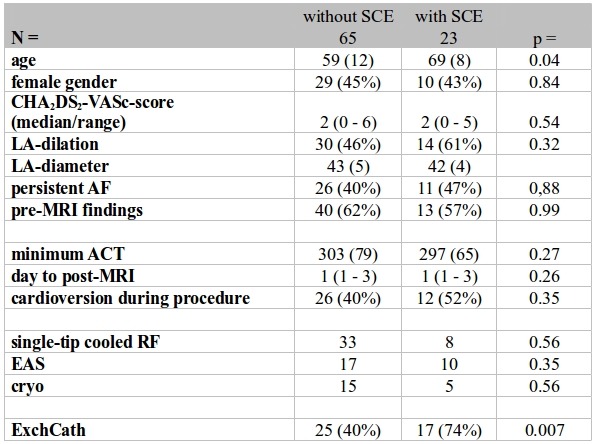
Univariate comparison of baseline and procedural characteristics between the group of patients without to those with SCE

**Table 3:**
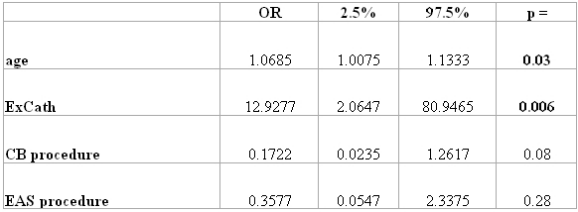
Risk for SCE assessed by multiple logistic regression
